# How can the audiogram be more useful?

**DOI:** 10.1186/s40463-016-0132-8

**Published:** 2016-03-10

**Authors:** Robert V Harrison

**Affiliations:** Department of Otolaryngology – Head & Neck Surgery, The Hospital for Sick Children, 555 University Ave., Toronto, ON M5G1X8 Canada

**Keywords:** Behavioral audiogram, High frequency hearing loss

## Abstract

The Canadian Hearing and Auditory Research Translation (CHART) group is a newly formed taskforce to develop collaborative research initiatives. Initial discussions centered on diagnostic improvements for middle ear disease, auditory neuropathy spectrum disorder, tinnitus and presbycusis. Central to these discussions was the widely held view that the standard audiogram and its interpretation is inadequate to for describing many forms of hearing problems that we now recognize. This letter is designed to create awareness and to seek feedback from hearing healthcare professionals on their experience regarding the adequacy (or otherwise) of the audiogram in its present form.

## Correspondence/Findings

### ‘Letters to the Editor’

A new Canada-wide initiative in for research collaborations in audiology/otology was recently (Nov. 2015) established. This is the Canadian Hearing and Auditory Research Translation (CHART) group. The Hearing Foundation of Canada (THFC) aided with some industry sponsorship (Oticon Foundation) held the first workshop in Halifax NS, hosted by Drs. Manohar Bance and Steve Aikin. Leaders in audiology, otology and hearing science from across the country discussed priority research questions, and plans to address them. The focus was on clinical problems rather than basic science issues. Four key areas were chosen, namely middle ear function, auditory neuropathy, tinnitus and presbyacusis.

In addition, and the reason for this note to clinicians, is that we also discussed the limitations of clinical diagnostic assessments for patients with hearing difficulties. Importantly we are of the opinion that the standard audiogram, and its interpretation is inadequate to address many forms of hearing problems that we now recognize.

Here are three examples:

[1] We have long known that threshold measures of hearing (i.e. the audiogram) very often do not correlate with real hearing difficulty. This notion was put into sharp focus when we formally recognized auditory neuropathy spectrum disorder (ANSD). More recently we have become aware of “hidden hearing loss”, from studies showing that noise exposure can deplete more auditory neurons than cochlear haircells. Cochlear thresholds can look normal despite the retro-cochlear lesions. Standard audiometry has no chance of revealing any subtle threshold deficits. However if we used a higher definition testing with 1–2 dB accuracy instead of 5–10 dB approximations, we might identify subtle clues about hidden hearing loss and ANSD problems.

[2] Why do we still, in 2016, not measure the whole high-frequency range to our hearing? Why do we only measure up to 8 kHz when we know that most hearing problems typically start in the high frequencies? Ototoxic drug damage, presbycusis, noise trauma can all cause hearing loss at frequencies above those that we routinely test. Some studies in patients with tinnitus that appear to have “normal audiograms” turn out to have very high frequency hearing loss (above 8 kHz), but not usually detected. We need to make high frequency audiometric testing a standard clinical procedure.

[3] Given the lack of level definition and frequency range of standard audiometry the interpretation of the audiogram for reporting purposes is very misleading. To suggest that a patient with threshold elevations of 10–15 dB is in the “normal range” is not useful. Statistically, a 5 dB threshold loss is significant! To report that a subject has “normal hearing” when at 16 kHz there may be an unmeasured 50 dB threshold elevation is clearly inaccurate. Our recognition of ANSD and “hidden hearing loss” etc. should, by now, have prompted us to revise our standard audiometric procedures. We need to see some evolution, and that will only come when audiologists and otologists agree that change is necessary.

Our CHART group would be very interested to hear your feedback comments on this audiogram issue. Please feel free to contact the group (through myself at: rvh@sickkids.ca).

On behalf of the CHART group

Robert V. Harrison

Professor, and Vice-Chair (research)

Department of Otolaryngology – Head & Neck Surgery

University of Toronto.

Director, Auditory Science Laboratory,

Program in Neuroscience and Mental Health

The Hospital for Sick Children, Toronto

CANADA

## Abbreviations

ANSD: Auditory neuropathy spectrum disorder
CHART: Canadian Hearing and Auditory Research Translation group
THFC: The Hearing Foundation of Canada

